# The Zinc-Finger protein ZCCHC3 inhibits LINE-1 retrotransposition

**DOI:** 10.3389/fmicb.2022.891852

**Published:** 2022-10-05

**Authors:** Zixiong Zhang, Ning Zhang, Saisai Guo, Qian Liu, Shujie Wang, Ao Zhang, Dongrong Yi, Jianyuan Zhao, Quanjie Li, Jing Wang, Yongxin Zhang, Ling Ma, Jiwei Ding, Shan Cen, Xiaoyu Li

**Affiliations:** Institute of Medicinal Biotechnology, Chinese Academy of Medical Sciences and Peking Union Medical School, Beijing, China

**Keywords:** ZCCHC3, LINE-1, retrotransposon, restricting factor, RNA

## Abstract

Long-interspersed element 1 (LINE-1) is an autonomous non-LTR retrotransposon. Its replication can cause mutation and rearrangement of host genomic DNA, which may result in serious genetic diseases. Host cells therefore developed defense strategies to restrict LINE-1 mobilization. In this study, we reported that CCHC-type zinc-finger protein ZCCHC3 can repress LINE-1 retrotransposition, and this activity is closely related to its zinc-finger domain. Further studies show that ZCCHC3 can post-transcriptionally diminish the LINE-1 RNA level. The association of ZCCHC3 with both LINE-1 RNA and ORF1 suggests that ZCCHC3 interacts with LINE-1 RNP and consequently causes its RNA degradation. These data demonstrate collectively that ZCCHC3 contributes to the cellular control of LINE-1 replication.

## Introduction

Long-interspersed element 1 (LINE-1) is a non-LTR (long terminal repeat) retrotransposon that can move its sequences from one site in the genome to the other through an RNA intermediate (Goodier and Kazazian, 2008). The human genome contains more than 500,000 copies of LINE-1 among which ~80–100 copies are capable of retrotransposition (Brouha et al., 2003). Of all transposons in the human genome, including DNA transposons, LTR retrotransposons, and non-LTR retrotransposons, including LINEs and SINEs (short interspersed elements), LINE-1 is the only active autonomous transposon in the human genome. Moreover, LINE-1 also supports the retrotransposition of SINES including Alu and SVA that do not encode their own proteins (Goodier and Kazazian, 2008; Richardson et al., 2015).

The full-length LINE-1 transcript is ~6,000 nucleotides in length, which includes a 5′ untranslated region (5′ UTR) harboring an active promoter, two open reading frames (ORF1p and ORF2p), and a 3′ UTR (Kazazian and Moran, 2017). ORF1p is a 40-kDa RNA-binding protein bearing nucleic acid chaperone activity (Martin, 2006, 2010; Martin et al., 2008), and ORF2p is a 150-kDa protein with endonuclease and reverse transcriptase activities (Feng et al., 1996; Alisch et al., 2006). Both ORF1p and ORF2p proteins show a strong cis-preference, and they associate with the LINE-1 RNA to form a ribonucleoprotein particle (RNP) in the cytoplasm (Kulpa and Moran, 2006). LINE-1 RNP is then imported into the nucleus, where the LINE-1 RNA is reverse transcribed into cDNA and subsequently incorporated into the host genome through a process termed target-primed reverse transcription (TPRT) (Luan et al., 1993; Cost et al., 2002).

The transposition of LINE-1, Alu, and SVA can cause genetic instability and induce dozens of genetic diseases (Volkman and Stetson, 2014; Hancks and Kazazian, 2016; Burns, 2017; Mita et al., 2020). To avoid the deleterious effects of LINE-1 transposition on the structure and function of the host genome, various strategies have been described to safeguard the host against LINE-1. One mechanism involves DNA methylation and histone modification, which suppresses LINE-1 RNA transcription (Garcia-Perez et al., 2010; Goodier, 2016; Ishak et al., 2016; Fukuda and Shinkai, 2020). Noncoding RNA (ncRNA)-based RNA interference machinery, including siRNA, miRNA, and piRNA, represent another controlling mechanism against these transposable elements (TEs) (Obbard et al., 2009; Newkirk et al., 2017; Teixeira et al., 2017; Fung et al., 2019). Moreover, in recent years, many interferon-inducible host proteins, such as APOBEC3 proteins, MOV10, SAMHD1, and ZAP, also have been found to process inhibitory activities against LINE-1 retrotransposition (Yu et al., 2015; Goodier, 2016).

The CCHC-type zinc-finger protein ZCCHC3 is a CCHC-type zinc-finger protein, which was recently discovered to involve in antiviral innate immune responses. Specifically, ZCCHC3 promotes the interaction between viral nuclear acid and pattern recognition receptors, including cGAS, RIG-I-like receptor, and Toll-like receptor 3, thus positively regulating IFN signaling triggered by RNA and DNA virus (Lian et al., 2018a,b; Zang et al., 2020). Besides, ZCCCH3 was also found to interact with SARS-CoV-2 Nucleocapsid Protein (Zheng et al., 2021), indicating its multifunctional roles in antiviral response. Endogenous retroelement LINE-1 is quite similar to exogenous viruses, which can still replicate itself in our genome and has also been reported to trigger innate immune responses. Therefore, it might be interesting to identify whether ZCCCH3 also involves in LINE-1 retrotransposition.

## Materials and methods

### Plasmids and antibodies

The LINE-1-firefly-luciferase reporter pWA367 and LINE-1-5′UTR promoter-luciferase reporter L1-FL were described earlier (Esnault et al., 2000; Athanikar et al., 2004; Xie et al., 2011). The Myc-ORF1p, Flag-ZCCHC3, and Flag-ZCCHC3-dc truncation cDNA sequences were cloned into the pcDNA3.0 expression vector (Invitrogen) by standard molecular biology techniques.

Flag-tag (D6W5B) Rabbit mAb (14793), Flag-tag (9A3) Mouse mAb (8146), Myc-Tag (71D10) Rabbit mAb (2278), Myc-Tag (9B11) Mouse mAb (2276), LINE-1 ORF1p (D3W9O) Rabbit mAb (88701), and ZCCHC3 Rabbit antibody (65321) were purchased from the Cell Signaling Technology. Flag-tag Goat antibody (ab95045) was purchased from Abcam. LINE-1-ORF1p Rabbit polyclonal antibody was a kind gift from Fei Guo (Hu et al., 2015). G3BP1 (H-10) Mouse mAb (sc-365338) was purchased from Santa Cruz.

### Cell culture and transfection

The human embryonic kidney 293T cells and HeLa cells were purchased from ATCC and were grown in DMEM supplemented with 10% fetal bovine serum (Invitrogen). Cells were transfected with Lipofectamine (Invitrogen) and VigoFect (Vigorous Biotechnology) according to the manufacturer's instructions.

### LINE-1 retrotransposition assay

Cells were seeded in six-well plates and transected with pWA367 plasmid and the Relina luciferase control plasmid pGL4.73 with a ratio of 20:1 and ZCCHC3 or empty vector reporter DNA. Twenty-four hours after transfection, puromycin (Sigma) was added in a complete medium at a 2.5 mg/ml final concentration. Cells were harvested 4 days post-transfection and lysed with Cell Culture Lysis Reagent (Promega). Luminescence was measured using the Dual-Luciferase Reporter Assay System (Promega) following the manufacturer's instructions.

### LINE-1 promoter-luciferase assay

Cells were seeded in six-well plates 1 day before transfection with 1 ug of the LINE-1 promoter-Luc reporter L1-FL and ZCCHC3 or empty vector reporter DNA. Forty-eight hours after transfection, cells were lysed with 5 × Cell Culture Lysis Reagent (Promega), and luciferase activity was quantified using Promega Luciferase Assay Kit (Promega) on a Berthold microplate reader.

### Quantitative real-time–PCR

Cells were transected with pWA367 and ZCCHC3 or empty vector reporter DNA. For detecting LINE-1 mRNA, cells were harvested 2 days post-transfection for analysis, and total RNA was extracted with TRIzol reagent (Invitrogen). An equal amount of total cellular RNA was treated with DNase to remove the potential contamination DNA and reverse transcribed with M-MLV Reverse Transcriptase (Promega) using the oligo dT primer. Q-PCR was performed using the SYBR Green Master Mix (Roche) and the ABI Quant-Studio 1. For detecting the LINE-1 integration, genomic DNA was extracted using QIAamp DNA Blood Mini Kit (Qiagen) 4 days post-transfection. Q-PCR was performed using Premix Ex Taq™ (Takara) and the ABI Quant-Studio 1. The following primer was used in detecting LINE-1, IFNB1, CXCL10, and the housekeeping gene GAPDH (Xie et al., 2011).

GAPDH forward 5-CCCACTCCTCCACCTTTGAC-3,
GAPDH reverse 5-TGTTGCTGTAGCCAAATTCGTT-3,
GAPDH probe 5-AAGCTCATTTCCTGGTATGA-3;
Fluc forward 5-GCAAAAGAAGCTACCGATCATACA-3,
Fluc reverse 5-GAAGCTCTCGGGCACGAA-3,
Fluc exon-exon junction probe 5-CTTCCCACCTGCCACC-3.
IFNB1 forward 5-TTGTTGAGAACCTCCTGGCT-3
IFNB1-reverse 5-TGACTATGGTCCAGGCACAG-3
CXCL10 forward 5-GGTGAGAAGAGATGTCTGAATCC-3
CXCL10 reverse 5-GTCCATCCTTGGAAGCACTGCA-3

### Western blotting

Cells were lysed in RIPA buffer [0.1% SDS, 1% Triton X-100, 1% sodium deoxycholate, 150 mM NaCl, 10 mM Tris (pH 7.5), and 1 mM EDTA]. Equal amounts of cell lysates were separated in SDS-10% PAGE. Proteins were transferred onto PVDF membranes (Whatman), blocked in 5% milk in TBST buffer, and then probed with antibodies as indicated. The same samples were used to detect the bands of different proteins using different gels in one panel because the molecular weights of ZCCHC3 (~46 kD), ORF1p (~40 kD), and Tubulin (~55 kD) are close.

### Co-immunoprecipitation

Cells were transfected with plasmids expressing FLAG-tagged ZCCHC3 and pWA367 or Myc-tagged LINE-1 ORF1p. Forty-eight hours after transfection. The transfected cells were lysed with NETN buffer (20 mM Tris–HCl, pH 8.0, 100 mM NaCl, 1 mM EDTA, and 0.5% NonidetP-40) and supplemented with a protease inhibitor cocktail (Roche). Insoluble material was pelleted at 13,000 g for 30 min, followed by RNase (Thermo) or not for 30 min at 37°C. Equal amounts of supernatant were incubated with 5 ul of anti-flag antibody (Abcam, mouse) or anti-Myc antibody (Abcam, mouse) for 16 h at 4°C, followed by the addition of protein A+G Sepharose (Beyotime Biotechnology) for 2 h. Beads were washed three times with NTEN buffer, boiled in 2 × SDS loading buffer, followed by Western blot analysis.

### RNA immunoprecipitation

Cells were transfected with 1,000 ng pWA367 with 500 ng ZCCHC3 DNA or empty vector. The cells were collected 48 h post-transfection and then lysed in 350 μl of TNT buffer (20 mM Tris. HCl, pH 7.5, 200 mM NaCl, and 1% Triton X-100) with RNase inhibitor (Takara) at a final concentration of 1 U/μl. The expressed ZCCHC3 was immunoprecipitated with an anti-flag antibody as described above. The RNA associated with the precipitated complex was extracted with TRIzol agents (Invitrogen) and subjected to RT–PCR using primers that amplify the luciferase as described above.

### Immunofluorescence microscopy

Cells were transfected with plasmids expressing FLAG-tagged ZCCHC3 and pWA367 or Myc-tagged LINE-1 ORF1p. Forty-eight hours after transfection. The cells were fixed with 4% paraformaldehyde (in 1phosphate-buffered saline) for 10 min at room temperature and permeabilized with 4% paraformaldehyde and 0.1% Triton X-100 for 10 min at room temperature. The cells were then stained for 2 h at room temperature with antibodies against FLAG (1:500 dilution, mouse) and Myc (1:500 dilution, rabbit). After being washed with 1 phosphate-buffered saline, the cells were incubated with either Alexa Fluor 488-conjugated secondary anti-mouse antibody or Alexa Fluor 594-conjugated secondary anti-rabbit antibody (1:2,000 dilution; Invitrogen). The images were recorded using the Zeiss Pascal laser scanning confocal microscope.

### siRNA knockdown

siRNA against ZCCHC3 (final concentration 50 nM) or non-targeting control (JTS scientific) was transfected to 293T cells using Lipofectamine RNAiMax (Invitrogen) according to the manufacturer's protocol. Forty-eight hours after the transfection, cells were used for experiments.

### Statistical analysis

All data are presented as means ± standard deviations (SD). The significance of differences is indicated in the figures. Statistical analyses were performed with a two-tailed, unpaired Student's *t*-test, using the GraphPad Prism software (^*^P < 0.05; ^**^P < 0.01; ^***^P < 0.001; ns., not significant.).

## Results

### ZCCHC3 inhibits LINE-1 retrotransposition

**Figure 1:**
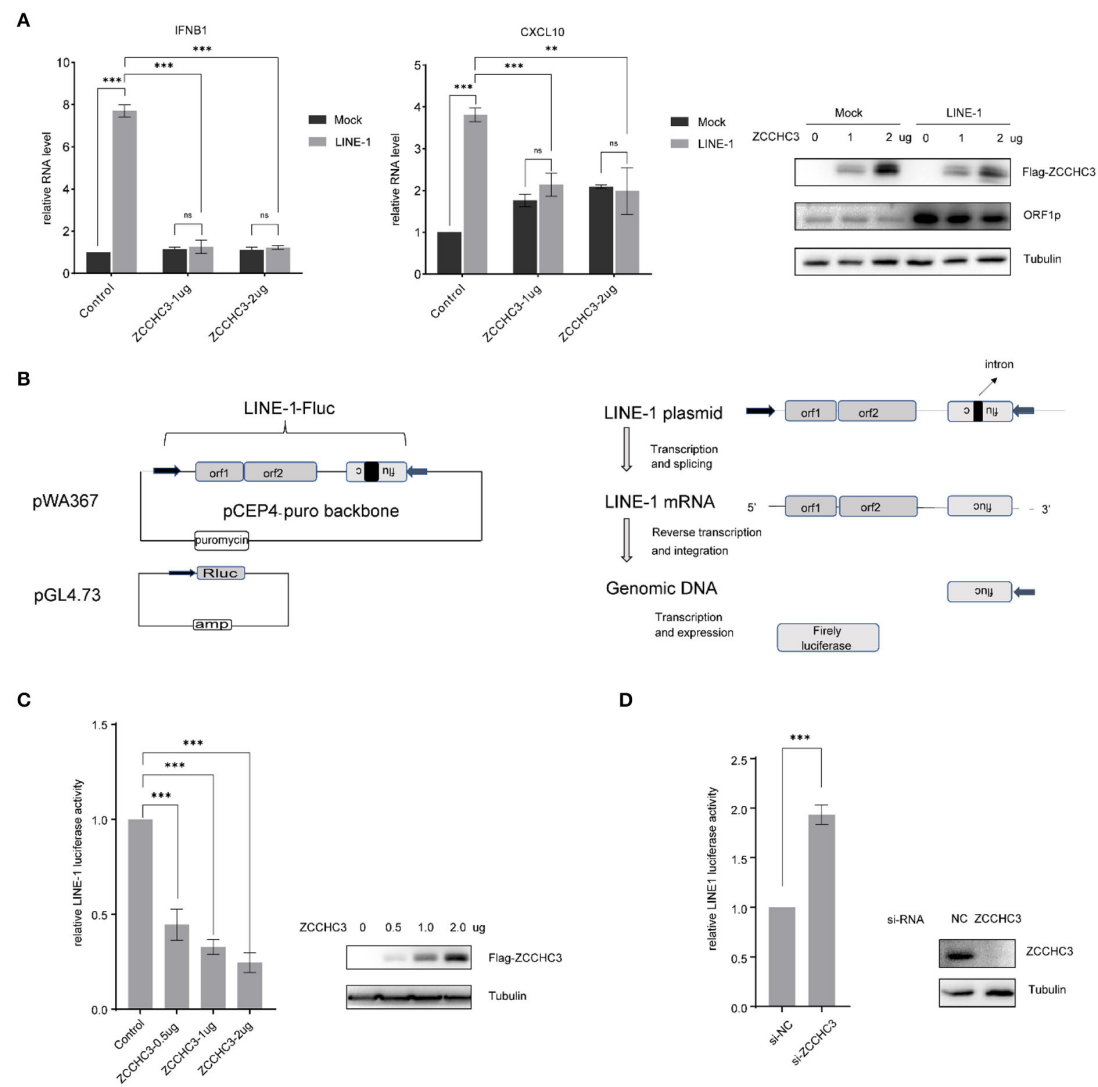
ZCCHC3 inhibits LINE-1 retrotransposition. **(A)** ZCCHC3 inhibits LINE-1-induced IFN production. HEK293T cells were transfected with pWA367 and ZCCHC3 DNA. Two days post-transfection, total RNA was extracted for IFNB1 (left panel) and CXCL10 RNA analysis using real-time PCR, and the RNA level was normalized to GAPDH (medial panel). Expression of the LINE-1 ORF1p and Flag-ZCCHC3 were analyzed by immunoblot, and Tubulin was detected as an internal control (right panel). **(B)** Diagrams of the LINE-1 reporter pWA367 plasmid and Rluc plasmid. pWA367 bears an intron-containing firefly luciferase gene in its 3′ UTR. This design ensures that the firefly luciferase will only be produced from the reverse transcribed LINE-1 DNA in the host genome, in which the intron should have been removed during RNA splicing. **(C)** Overexpression of ZCCHC3 inhibits LINE-1 retrotransposition. HEK293T cells were transfected with pWA367, pGL4.73, and either empty vector or increasing amounts of vector encoding ZCCHC3. Four days post-transfection, cell lysates were analyzed for luminescence activity (left panel). Expression of the Flag-ZCCHC3 was analyzed by immunoblot, and Tubulin was detected as an internal control (right panel). **(D)** Knockdown of ZCCHC3 increases LINE-1 retrotransposition. HEK293T cells were transfected with pWA367, pGL4.73, and the siRNA targeting ZCCHC3. Four days post-transfection, cell lysates were analyzed for luminescence (left panel). Expression of the ZCCHC3 was analyzed by immunoblot, and Tubulin was detected as an internal control (right panel). Data are representative of at least three independent experiments, values are expressed as means ± SD, and representative blots and images are shown.

As ZCCCH3 was reported to positively regulate virus-triggered IFN production, we first investigated whether ZCCHC3 also plays a role in the LINE-1-induced IFN pathway, which was believed to contribute to the auto-immune response under the circumstance of non-infection of the exogenous virus (Volkman and Stetson, 2014; De Cecco et al., 2019; Saleh et al., 2019; Simon et al., 2019). We transfected LINE-1 DNA with different amounts of ZCCHC3 expression plasmid into the HEK293T cells, and then detected the ability of ZCCHC3 to regulate the LINE-1-induced IFN pathway. Unexpectedly, ectopically expressed ZCCHC3 decreased the production of LINE-1 stimulated Interferon Beta1 (IFNB1) and interferon-γ-inducible protein 10(CXCL10) significantly, and it seems that the expression of 1 ug ZCCHC3 plasmid has reached its saturation concentration for affecting IFNB1 or CXCL10 level in cells, and the effect of more ZCCHC3 DNA(2ug) transfection is not obvious (Figure 1A). Collectively, these data indicated that ZCCHC3 negatively regulates the LINE-1-triggered innate response.

It has been reported that cytoplasmic LINE-1 RNA and cDNA can trigger the IFN-I response (Lagisquet et al., 2021). We therefore speculated that ZCCHC3 may repress LINE-1 elements replication and retrotransposition, which might directly diminish the accumulation of RNA or cDNA derived from LINE-1 elements, thus diminishing LINE-1-triggered innate response. To investigate the effect of ZCCHC3 on LINE-1 retrotransposition, we utilized the established LINE-1 luciferase reporter pWA367 (Xie et al., 2011), which bears an intron-containing firefly luciferase gene in its 3′ UTR. This design ensures that the firefly luciferase will only be produced from the reverse transcribed LINE-1 DNA in the host genome, in which the intron should have been removed during RNA splicing, and therefore, the luciferase activity reflects the events of LINE-1 retrotransposition. Furthermore, a control plasmid encoding Rluc was co-transfected to normalize the expression of the firefly luciferase gene (Figure 1B).

We transfected the pWA367 plasmid with or without ZCCHC3 DNA into the HEK293T cells, followed by a luciferase activity detection assay. Although we did not observe any significant effect of ZCCHC3 ectopic expression on the viability of cells or the Rluc activity, which most likely excludes the possibility that ZCCHC3 might impair luciferase expression or lead to cellular cytotoxicity, the transient expression of ZCCHC3 diminished the luciferase activity by ~50, 70, and 80% (*p* < 0.05), according to different ZCCHC3 expression levels (Figure 1C), indicating that overexpression of ZCCHC3 can inhibit LINE-1 retrotransposition in a dose-dependent manner. We next depleted the endogenous ZCCHC3 in HEK293T cells using siRNA oligos to investigate how LINE-1 activity was affected. The activity of luciferase which represents LINE-1 retrotransposition increased **~2**-fold when ZCCHC3 was depleted with siRNA (Figure 1D), suggesting that endogenous ZCCHC3 also has the LINE-1 inhibitory activity. Together, these data indicated that ZCCHC3 can restrict LINE-1 retrotransposition.

### ZCCHC3 reduces the LINE-1 insertion

**Figure 2:**
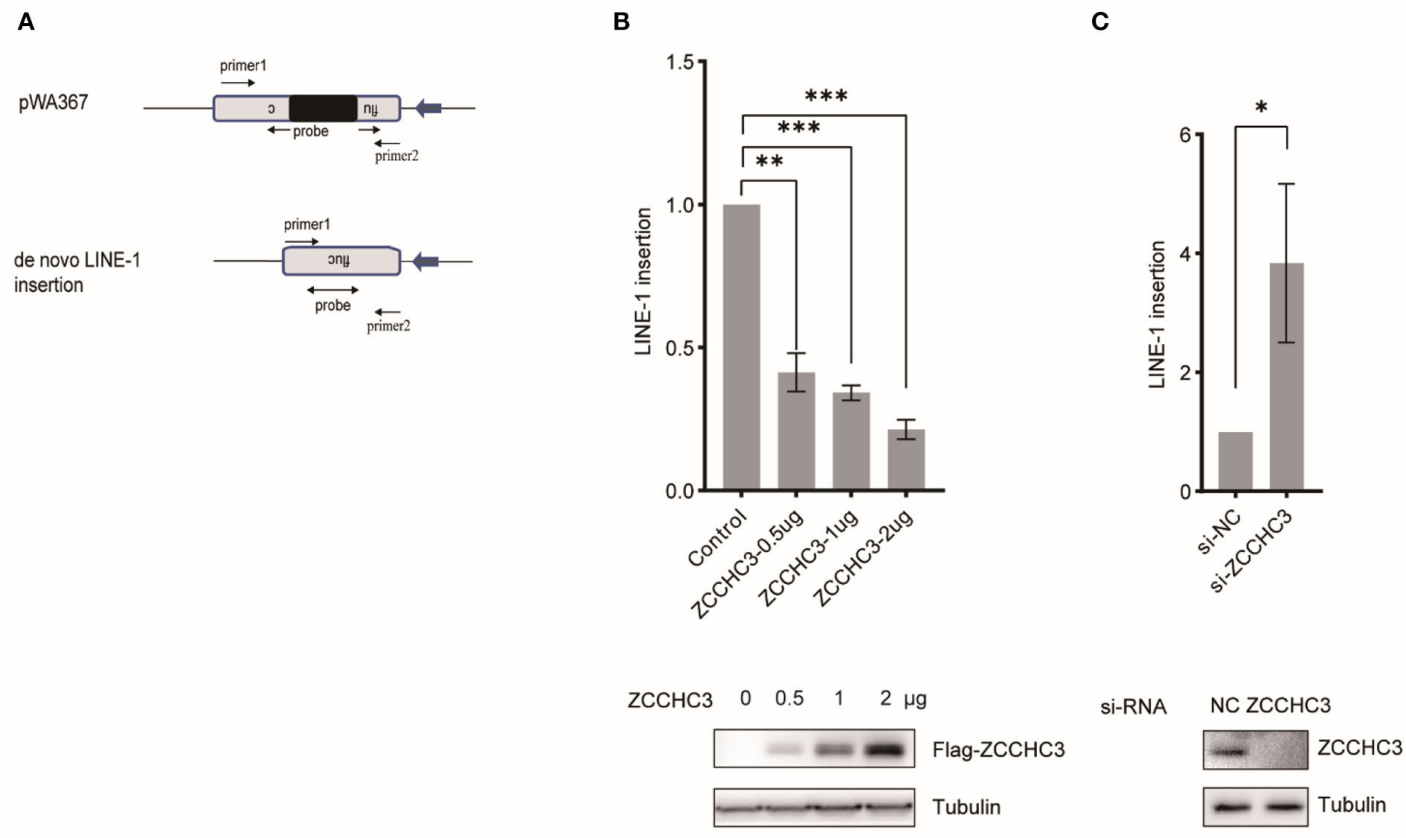
ZCCHC3 decreased LINE-1 insertions. **(A)** Diagrams of Taqman real-time PCR to detect novel LINE-1 integrates. The real-time PCR was performed with two primers and one probe across the intron to detect the integrated novel LINE-1. **(B)** Overexpression of ZCCHC3 decreased novel LINE-1 integrates. HEK293T cells were transfected with pWA367 and ZCCHC3 DNA. Four days post-transfection, genomic DNA was extracted and analyzed for Taqman real-time PCR (top panel). Expression of the Flag-ZCCHC3 was analyzed by immunoblot, and Tubulin was detected as an internal control (bottom panel). **(C)** Knockdown of ZCCHC3 increased novel LINE-1 integrates. HEK293T cells were transfected with pWA367 together with the siRNA targeting ZCCHC3. Four days post-transfection, genomic DNA was extracted and analyzed for Taqman real-time PCR (top panel). Expression of the ZCCHC3 was analyzed by immunoblot, and Tubulin was detected as an internal control (bottom panel). Data are representative of at least three independent experiments, values are expressed as means ± SD, and representative blots and images are shown.

To assess LINE-1 repression by ZCCHC3 independently of reporter gene expression, we imposed TaqMan probe real-time PCR to determine the relative copy number of the novel LINE-1 integrates, which were quantified in triplicate with primers and an exon-exon junction probe specific to the firefly luciferase gene to confirm the intron-removal form of LINE-1 insertions (Figure 2A). The result showed that ectopically expressed ZCCHC3 significantly reduced the copy number of the non-intron luciferase gene (Figure 2B), while knocking down the endogenous ZCCHC3 increased the LINE-1 insertion ~3-fold (Figure 2C), thus confirming the repression of LINE-1 by ZCCHC3 independently of luciferase reporter gene expression

### ZCCHC3 decreases LINE-1 RNA level

**Figure 3:**
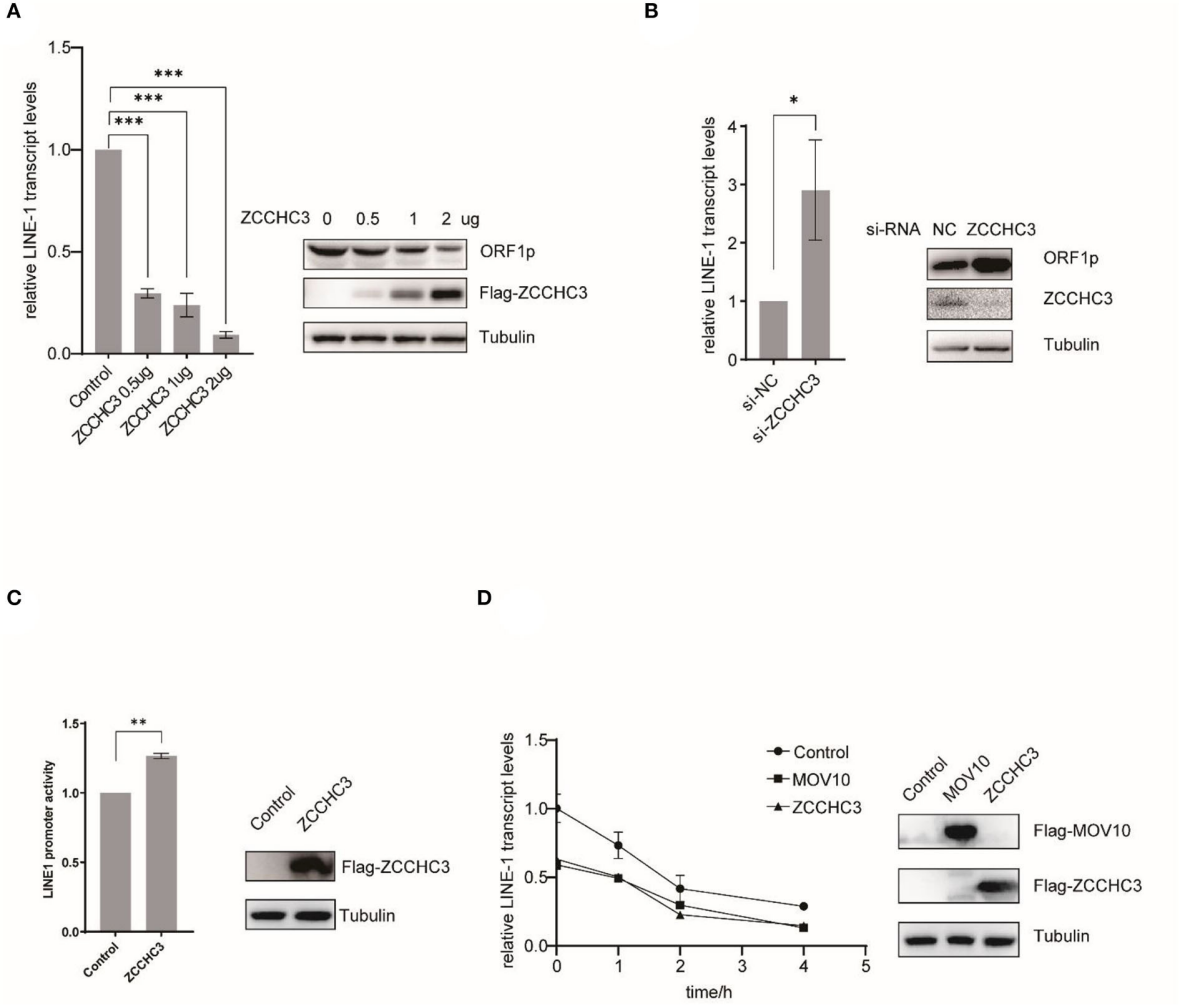
ZCCHC3 diminished LINE-1 RNA and ORF1p. **(A)** Overexpression of ZCCHC3 decreases LINE-1 RNA and ORF1p. HEK293T cells were transfected with pWA367 and ZCCHC3 DNA. Total RNA was extracted for Taqman real-time PCR analysis, the RNA level was normalized to GAPDH (left panel). Expression of the LINE-1 ORF1p and Flag-ZCCHC3 were analyzed by immunoblot, and Tubulin was detected as an internal control (right panel). **(B)** Knockdown of ZCCHC3 increases LINE-1 RNA and ORF1p level. HEK 293T cells were transfected with pWA367 together with the siRNA targeting ZCCHC3. Total RNA was extracted for Taqman real-time PCR analysis, the RNA level was normalized to GAPDH (left panel). Expression of the LINE-1 ORF1p and ZCCHC3 were analyzed by immunoblot, and Tubulin was detected as an internal control (right panel). **(C)** ZCCHC3 does not affect the activity of the LINE-1 5′UTR promoter. HEK293T cells were transfected with the L1-FL reporter plasmid and ZCCHC3 DNA. Cell lysates were analyzed for luminescence (left panel). Expression of the ZCCHC3 was analyzed by immunoblot, and Tubulin was detected as an internal control (left panel). **(D)** The detection of RNA stability. HEK293 cells were co-transfected with pWA367 and either ZCCHC3, MOV10 DNA, or pcDNA3.0. Twenty-four hours post-transfection, the cells were treated with actinomycin D, and then detected LINE-1 RNA levels at various times as indicated by Taqman real-time PCR, the RNA level was normalized to GAPDH (left panel). Expression of the ZCCHC3 and MOV10 were analyzed by immunoblot, and Tubulin was detected as an internal control (left panel). Data are representative of at least three independent experiments, values are expressed as means ± SD, and representative blots and images are shown.

We next set up to investigate in which step of the LINE-1 life cycle ZCCHC3 plays a crucial role. For LINE-1 RNA both serve as mRNA for protein expression and templates to produce new cDNA copies. LINE-1 RNA levels affected the copy numbers of LINE-1 DNA. We therefore next examined the effect of ZCCHC3 on LINE-1 RNA. We transfected pWA367 plasmid with or without ZCCHC3 DNA into the HEK293T cells, and then detected the effect of ectopically expressed ZCCHC3 on LINE-1 RNA using RT–qPCR. The result showed that the transient expression of ZCCHC3 can diminish the LINE-1 RNA level at a similar level as it inhibits LINE-1 retrotransposition, suggesting that the inhibitory activity of ZCCHC3 may be derived from diminishing LINE-1 RNA level (Figure 3A).

For LINE-1 RNA serves as mRNA for protein expression, we also detected the LINE-1 ORF1p expression level using immunoblotting and found that ZCCHC3 can reduce the LINE-1 ORF1p expression level as well (Figure 3A). Similarly, when we silenced the expression of endogenous ZCCHC3, LINE-1 RNA, and ORF1p levels increased ~2-fold (Figure 3B), indicating that both exogenous and endogenous ZCCHC3 can decrease LINE-1 RNA levels.

We speculated that ZCCHC3 decreases the LINE-1 RNA level might through affecting its transcription or influence its stability. We first examined whether ZCCHC3 affected LINE-1 RNA transcription. It has been reported that the 5′-UTR of LINE-1 serves as an internal promoter to drive LINE-1 RNA production (Goodier and Kazazian, 2008). We therefore used another reporter DNA construct, L1-FL, which only bears the LINE-1 5′-UTR sequence upstream of the firefly luciferase reporter gene (Athanikar et al., 2004) to detect the influence of the ZCCHC3 upon the LINE-1 internal promotor. We transfected this reporter DNA into HEK293T cells together with ZCCHC3 DNA, and then detected the luciferase activity. The presence of ZCCHC3 did not negatively affect the luciferase activity according to our results (Figure 3C), suggesting that ZCCHC3 did not influence LINE-1 RNA transcription driven by the 5′-UTR internal promoter, which indicates ZCCHC3 modulates LINE-1 RNA expression at the post-transcriptional stage. To test whether ZCCHC3 influences LINE-1 RNA stability, we used actinomycin D (5 μg/ml) to block the transcription from RNA polymerase II and measure the decay kinetics of LINE-1 RNA with overexpressed ZCCHC3. As shown in Figure 3D, the expression of ZCCHC3 diminished the amount of LINE-1 RNA to ~50% of that in the control cells at time 0. This phenomenon is similar to that of another host factor MOV10, which was reported to mediate the rapid degradation of LINE-1 RNA (Li et al., 2013; Choi et al., 2018). These results suggested that ZCCHC3 might also promote the rapid degradation of LINE-1 RNA as MOV10 did.

### ZCCHC3 interacts with LINE-1

**Figure 4:**
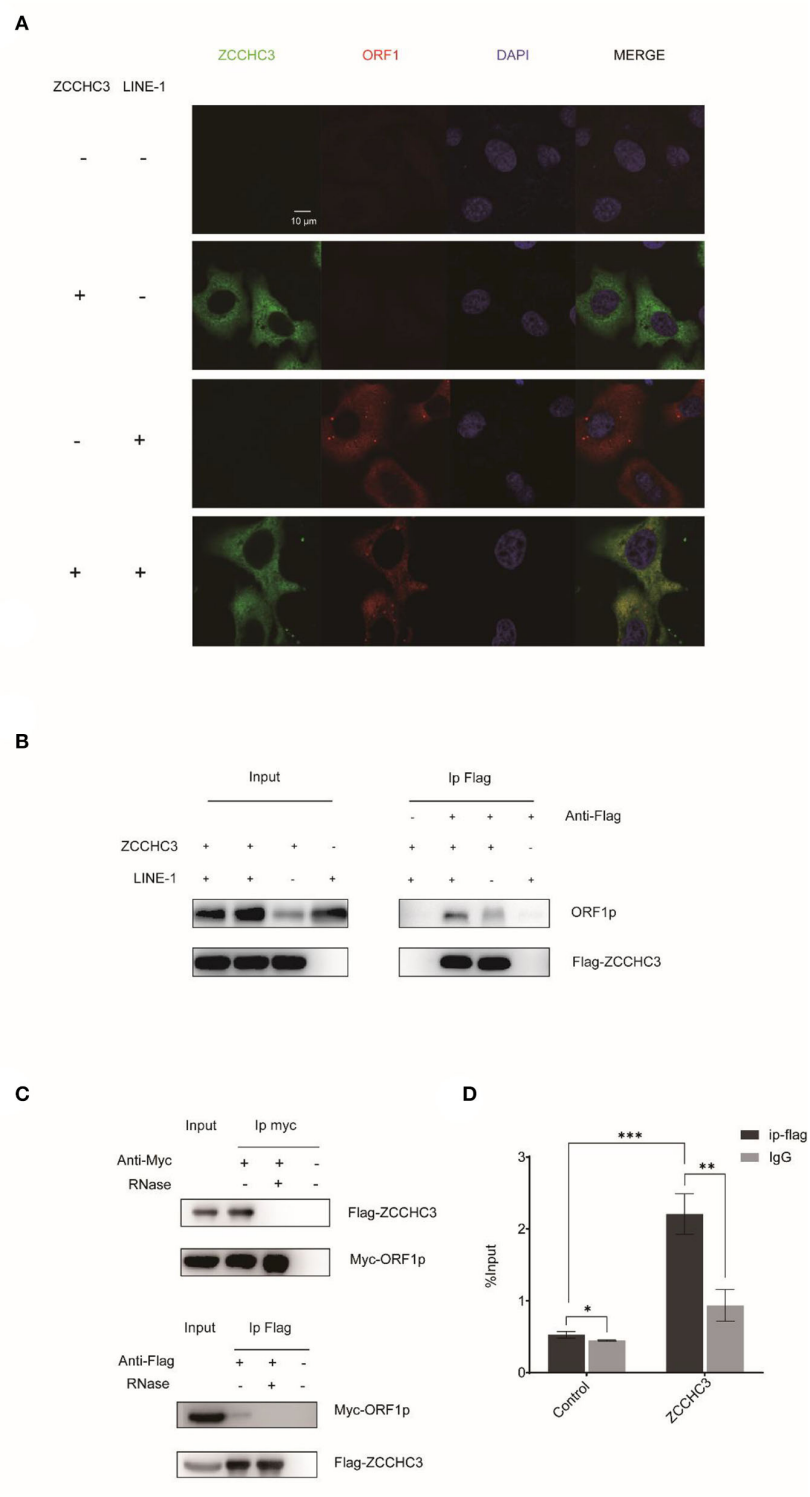
ZCCHC3 interacts with LINE-1 in the cytoplasm. **(A)** ZCCHC3 is co-located with LINE-1 ORF1p. Hela cells were transfected with pWA367 plasmid and Flag-ZCCHC3 DNA. Forty-eight hours post-transfection, LINE-1 ORF1p and Flag ZCCHC3 were detected by indirect immunofluorescence staining. **(B)** ZCCHC3 interacts with LINE-1 ORF1p. HEK293T cells were transfected with pWA367 plasmid and Flag-ZCCHC3 DNA. The cells were collected and lysed 48 h post-transfection. Equal amounts of supernatant were incubated with Flag antibody or lgG (mouse), LINE-1 ORF1p, and Flag-ZCCHC3 were analyzed by Western blots using antibodies against ORF1p and Flag (rabbit). **(C)** The interaction between ZCCHC3 and LINE-1 ORF1p is RNA-dependent. HEK293T cells were transfected with Flag-ZCCHC3 and Myc-ORF1p DNA. Forty-eight post-transfection, the cells were collected and lysed, and then treated with RNase at 37°C for 30 min, equal amounts of supernatant were incubated with Flag or Myc antibody (mouse) and then analyzed by Western blots using antibodies against Myc or Flag (rabbit). **(D)** ZCCHC3 binds to LINE-1 RNA. HEK293T cells were co-transfected with pWA367 DNA and ZCCHC3 DNA. Forty-eight hours later, an equal amount of cell lysis was used to detect LINE-1 RNA by RNA-IP. LINE-1 RNA pulled down by ZCCHC3 was quantified by RT–qPCR. These experiments were repeated three times and representative blots and images are shown.

ORF1p and ORF2p proteins associate with the LINE-1 RNA to form LINE-1 RNP in the cytoplasm. Although the role of LINE-1 RNP is still unclear, it protects LINE-1 RNA from degradation by different kinds of host machinery (Taylor et al., 2016). Therefore, it is tempting to speculate that ZCCHC3 might target the components of LINE-1 RNP to destabilize LINE-1 RNA. We therefore performed Immunofluorescence and co-immunoprecipitation (co-IP) to identify whether there is an association between LINE-1 and ZCCHC3. The image showed that ZCCHC3 was mainly presented in the cytoplasm, and co-located with LINE-1 ORF1p (Figure 4A). The co-IP results also indicated that ZCCHC3 is associated with LINE-1 ORF1p (Figure 4B). For LINE-1 ORF1p and ORF2p usually associate with the LINE-1 RNA as an RNP form in the cytoplasm, ZCCHC3 may interact directly with ORF1p or mediated by LINE-1 RNA. Indeed, the interaction between ZCCHC3 and LINE-1 ORF1p seems to be RNA dependent, for the interaction was lost after RNase treatment (Figure 4C), suggesting that the interaction between ZCCHC3 and ORF1p might be mediated by LINE-1 RNA. The RNA immunoprecipitation assay result also confirmed that there was an interaction between ZCCHC3 and LINE-1 RNA (Figure 4D). These results indicated that the interaction between ZCCHC3 and ORF1p was mediated by LINE-1 RNA.

### The Zinc-Finger domain is necessary to repress LINE-1 retrotransposition

**Figure 5:**
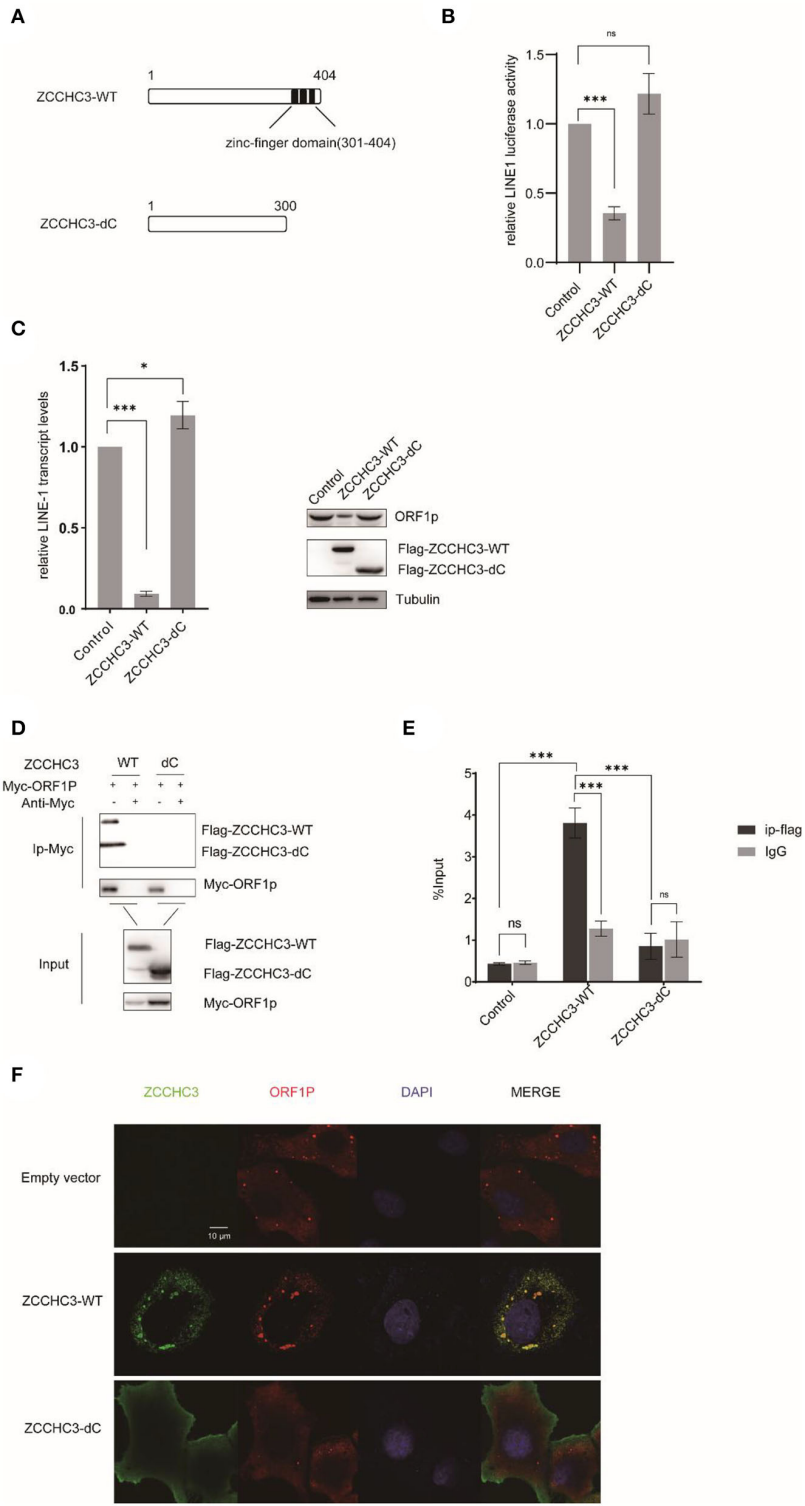
The zinc-finger domain is essential for ZCCHC3-mediated LINE-1 restriction. **(A)** The illustration of ZCCHC3 truncation. **(B)** ZCCHC3 dc does not inhibit LINE-1. HEK293T cells were transfected with pWA367 and either empty vector, Flag-ZCCHC3 or Flag-ZCCHC3-dc. 4 days post-transfection, cell lysates were analyzed for luminescence. **(C)** ZCCHC3 dc does not diminish LINE-1 RNA and ORF1p. Two days post-transfection, total RNA was extracted and LINE-1 RNA was normalized by the GAPDH and analyzed for Taqman real-time PCR (left panel). Expression of the LINE-1-ORF1p and Flag-ZCCHC3 were analyzed by immunoblot, and Tubulin was detected as an internal control (right panel). **(D)** ZCCHC3 dc does not interact with LINE-1 ORF1p. HEK293T cells were transfected with Myc-ORF1p and Flag-ZCCHC3 or Flag-ZCCHC3-dc. The cells were collected and lysed 48 h post-transfection. Equal amounts of supernatant were incubated with Myc antibody(mouse), Myc-ORF1p, and Flag-ZCCHC3 or Flag-ZCCHC3-dc and were analyzed by Western blots using antibodies against Myc and Flag(rabbit). **(E)** ZCCHC3 dc does not bind to LINE-1 RNA. HEK293T cells were co-transfected with pWA367 DNA and ZCCHC3-WT or ZCCHC3-dC DNA. Forty-eight hours later, an equal amount of cell lysis was used to detect LINE-1 RNA by RNA-IP. LINE-1 RNA pulled down by ZCCHC3 was quantified by RT–qPCR. **(F)** ZCCHC3 dc is not located with LINE-1 ORF1p. Hela cells were transfected with an empty vector or vector encoding Flag-ZCCHC3 or Flag-ZCCHC3-dc and Myc-ORF1p. Forty-eight hours post-transfection, Myc-ORF1p and Flag ZCCHC3 or Flag-ZCCHC3-dc were detected by indirect immunofluorescence staining. Data are representative of at least three independent experiments, values are expressed as means ± SD, and representative blots and images are shown.

The RNA-dependent interaction between ZCCHC3 and LINE-1 ORF1p suggested the potential role of RNA binding in LINE-1 repression by ZCCHC3. ZCCHC3 bears three tandem zinc finger domains in its c-terminal region (301-404aa), which was reported to have RNA binding activity (Lian et al., 2018a,b; Zang et al., 2020). We generated a c-terminal truncation mutant of ZCCHC3 and ZCCHC3-dc, which lacks the zinc-finger region, to elucidate whether the zinc-finger domains are necessary for its LINE-1 inhibitory function (Figure 5A). LINE-1 retrotransposition assay showed that ZCCHC3-dc almost completely lost the activity to restrict LINE-1 retrotransposition (Figure 5B). Correspondingly, ZCCHC3-dc also lost the activity to diminish LINE-1 mRNA and ORF1p protein in HEK293T cells (Figure 5C), indicating that the zinc-finger domain is important for ZCCHC3 to restrict LINE-1 elements. Furthermore, the co-IP and RIP results confirmed that ZCCHC3-dc lost the ability to interact with LINE-1 RNP (Figures 5D,E). Immunofluorescence results also showed that, although ZCCHC3-dc is still localized in the cytoplasmic, it lost the association with LINE-1 ORF1p in Hela cells (Figure 5F). To correspond to previous experiments, we also confirmed the same results in 293T cells (Supplementary Figure 1). We speculated the deletion of the zinc-finger made ZCCHC3 lose the ability to bind RNAs and appear in a more diffused distribution in the cytoplasm. These results suggest the important role of the zinc-finger domain for ZCCHC3 to interact with LINE-1.

## Discussion

ZCCHC3 possesses three tandem zinc finger domains in its C-terminal region. Zinc finger proteins are primarily considered to be DNA binding transcription factors, However, certain classes of zinc finger proteins can also interact with RNA, and are involved in the regulation of multiple steps of RNA metabolism, such as mRNA splicing, polyadenylation, transportation, translation, and decay (Fu and Blackshear, 2017; Wang et al., 2017). As a CCHC-type zinc finger protein, ZCCHC3 was recently reported to bind viral dsRNA and dsDNA and positively regulate virus-triggered induction of type I IFNs in cGAS, RIG-I, and TLR3-mediated innate immune responses (Lian et al., 2018a,b; Zang et al., 2020). However, in the absence of exogenous microbial infection, endogenous retroelements LINE-1 was believed to be a potential source of immunogenic cytoplasmic nucleic acids in the context of sterile inflammation. Cytoplasmic LINE-1 can trigger IFN-I response through RIG-I- and MDA5-mediated RNA sensing pathways (Zhao et al., 2018; Tunbak et al., 2020) and cGAS-mediated DNA sensing pathways (De Cecco et al., 2019; Simon et al., 2019; Gamdzyk et al., 2020), especially during cellular senescence, when LINE-1 become transcriptionally derepressed.

In this study, we first investigate the role of ZCCHC3 in the IFN-I response of LINE-1. According to our results, ZCCHC3 activated the IFN pathway, especially the expression of CXCL10, which corresponds with the results of Shu's reports (Lian et al., 2018a,b). But unexpectedly, ZCCHC3 negatively regulates LINE-1 triggered IFN production, which is different from the process of the exogenous virus. Our result indicated that ZCCHC3 represses the LINE-1 element retrotransposition by directly diminishing the RNA level of LINE-1. It is plausible that ZCCHC3 negatively regulates LINE-1 triggered IFN production by decreasing the LINE-1 cDNA and RNA levels, which are the source of triggering LINE-1 mediated IFN production.

The association between ZCCHC3 and LINE-1 was confirmed by the observation of the co-localization of ZCCHC3 with ORF1p in the cytoplasm and was further supported by the immunoprecipitation result. However, LINE-1 ORF1p and ORF2p are usually associated with the LINE-1 RNA as an RNP form in the cytoplasm, and the loss of the association between ZCCHC3 and ORF1p upon RNase treatment suggests that ZCCHC3 interact with LINE-1 ORF1p in an RNA-dependent manner, or ZCCHC3 directly bind the LINE-1 RNA but not ORF1p. On the other hand, zinc finger domains in ZCCHC3 were reported to have RNA binding activity. When we deleted the zinc finger domains of the ZCCHC3, it lost the association with LINE-1, further supporting the notion that ZCCHC3 might directly bind to LINE-1 RNA. Indeed, ZCCHC3 was reported to directly bind to viral dsDNA or dsRNA in previous studies, and the double-strand structure is necessary for the binding (Lian et al., 2018a,b). However, LINE-1 RNA is quite similar to cellular single-strand mRNA. It is worth investigating how ZCCCH3 binds to LINE-1 RNA in the future. A possible explanation would be the stem-loop structure in LINE-1 mRNA, which remains for further exploration.

Our result indicated that the inhibitory activity of ZCCHC3 may be derived from diminishing LINE-1 RNA, although the exact mechanism is still not clear. For immunofluorescence assay showed that ZCCHC3 is a cytoplasmic protein, which is unlikely to affect the transcription of LINE-1 in the nucleus. We also confirmed that ZCCHC3 did not influence LINE-1 RNA transcription driven by the 5′-UTR internal promoter. These results suggested a possibility that ZCCHC3 might induce LINE-1 RNA degradation in the cytoplasm. Indeed, the RNA stability analysis suggests a rapid degradation of LINE-1 RNA caused by ZCCHC3. This phenomenon is also observed in MOV10-induced RNA degradation (Li et al., 2013). We noticed that, upon overexpression of ZCCHC3, ORF1p formed large foci in the cytoplasm, in which ZCCHC3, ORF1p, and stress granule (SG) marker protein, G3bp1, were co-localized (Supplementary Figure 2), which suggests that ZCCHC3 causes sequestration of LINE-1 ORF1p in large stress granules. It is reported previously that endogenous or over-expressed ORF1p can spontaneously aggregate in SGs, in the absence of external stress (Goodier et al., 2007). Several host factors, such as Zinc-Finger Antiviral Protein (ZAP) (Gao et al., 2002), MOV10 (Goodier et al., 2012; Li et al., 2013), and The SAM domain and HD domain-containing protein 1 (SAMHD1) (Hu et al., 2015) were found to co-localize with LINE-1 RNP in SGs and induced the LINE-1 RNA degradation. In another study, Martin Brouha et al. also purified and identified 37 high-confidence host factors, including both MOV10 and ZCCHC3, form LINE-1 RNP complexes using affinity proteomics methods (Taylor et al., 2013). Based on the important role of SG in RNA metabolism, it is not surprising that these proteins co-localize in SGs with LINE-1 RNP and limited LINE-1 mobility. Stress granules might provide a platform to orchestrate the innate immune response to fight the exogenous and endogenous viruses attack, and the exact mechanism needs further investigation.

## Data availability statement

The raw data supporting the conclusions of this article will be made available by the authors, without undue reservation.

## Author contributions

Conceptualization: XYL, SC, and ZXZ. Performing the main part of the experiment: ZXZ and NZ. Methodology: ZXZ, SSG, and NZ. Software: QL. Validation: SSG, SJW, NZ, and AZ. Investigation: QJL, JW, and LM. Resources: JWD, DRY, JYZ, and YXZ. Writing—original draft preparation: ZXZ. Writing—review and editing: JWD. Supervision: XYL. Project administration: SC. Funding acquisition: XYL and SC. All authors have read and agreed to the published version of the manuscript.

## Funding

This research was funded by the CAMS Innovation Fund for Medical Sciences, CIFMS 2021-I2M-1-043 to XL and the National Natural Science Foundation of China Grant, 31870164.

## Conflict of interest

The authors declare that the research was conducted in the absence of any commercial or financial relationships that could be construed as a potential conflict of interest.

## Publisher's note

All claims expressed in this article are solely those of the authors and do not necessarily represent those of their affiliated organizations, or those of the publisher, the editors and the reviewers. Any product that may be evaluated in this article, or claim that may be made by its manufacturer, is not guaranteed or endorsed by the publisher.
