# Supplementary material for: The Zinc-Finger protein ZCCHC3 inhibits LINE-1 retrotransposition
Source: Front Microbiol. 2022 Oct 5;13:891852. doi: 10.3389/fmicb.2022.891852 (PMC9580041; doi:10.3389/fmicb.2022.891852)
Supplement: Supplementary file 1 [file Data_Sheet_1.docx]

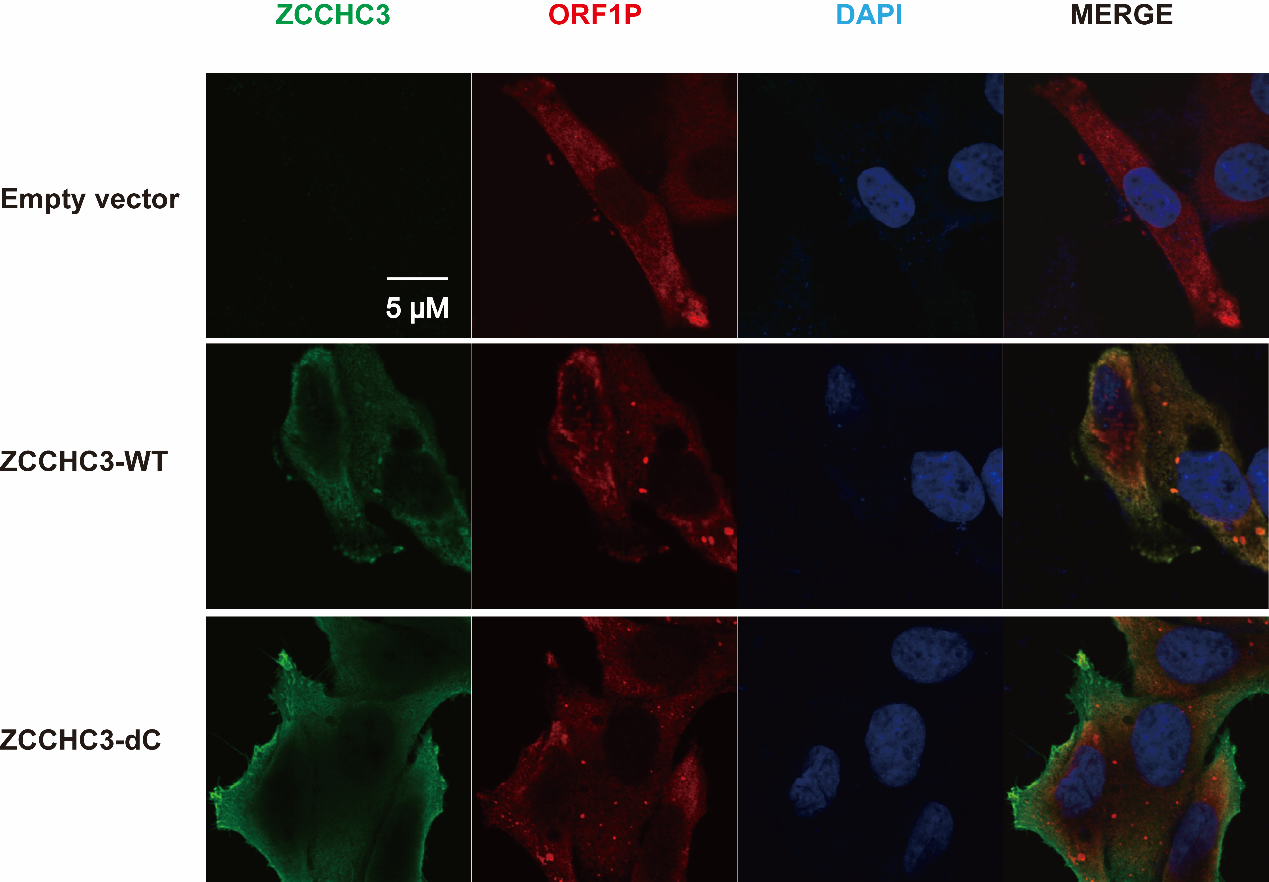


**Supplementary Figure 1. ZCCHC3 dc is not located with LINE-1 ORF1p.** 293T cells were transfected with an empty vector or vector encoding Flag-ZCCHC3 or Flag-ZCCHC3-dc and Myc-ORF1p. 48 hours post-transfection, Myc-ORF1p and Flag ZCCHC3 or Flag-ZCCHC3-dc were detected by indirect immunofluorescence staining. Data are representative of at least three independent experiments, and values are expressed as means ± SD and representative blots and images are shown.


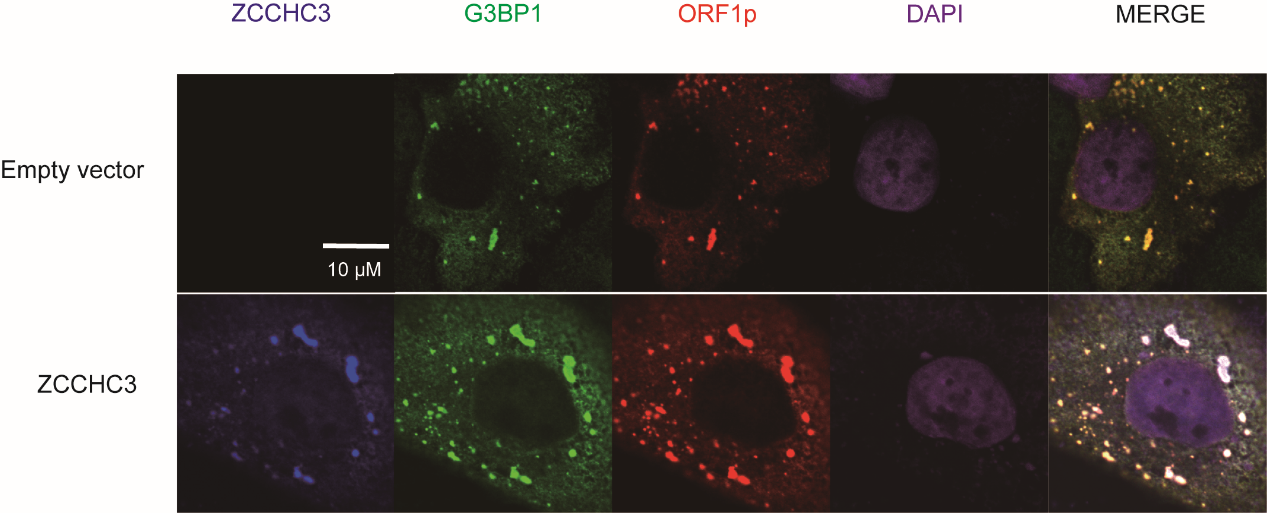


**Supplementary Figure 2. ZCCHC3 co-locates with G3BP1 and LINE1 ORF1p.** Hela cells were transfected with Myc-LINE1-OFR1p and vector encoding Flag-ZCCHC3 or not. 48 hours post transfection, LINE1 ORF1p, Flag ZCCHC3 and G3BP1 were detected by indirect immunofluorescence staining. Data are representative of at least three independent experiments, and values are expressed as means ± SD and representative blots and images are shown.
